# The innervation of the human acetabular labrum and hip joint: an anatomic study

**DOI:** 10.1186/1471-2474-15-41

**Published:** 2014-02-14

**Authors:** Abdullah Alzaharani, Kamal Bali, Ravi Gudena, Pamela Railton, Dragana Ponjevic, John R Matyas, James N Powell

**Affiliations:** 1 Orthopedic Trauma & Lower Extremity Reconstruction, 3134 Hospital Drive N.W, Calgary, Alberta T2N 5A1, Canada

**Keywords:** Labrum, Innervation, Femoro-acetabular impingement, Hip arthroscopy, Labral tear

## Abstract

**Background:**

The aim of the current study was to evaluate the innervation of the acetabular labrum in the various zones and to understand its potential role in nociception and proprioception in hips with labral pathology.

**Methods:**

A total of twenty hip labrums were tagged and excised intraoperatively from patients undergoing a total hip replacement. After preparation, the specimens were cut to a thickness of 10 μm and divided into four quadrants (zones) using a clock face pattern. Neurosensory structure distribution was then evaluated using Hematoxylin and Eosin (H and E), and immunoreactivity to S-100.

**Results:**

All specimens had abundant free nerve endings (FNEs). These were seen predominantly superficially and on the chondral side of the labrum. In addition, predominantly three different types of nerve end organs (NEOs) were identified in all twenty specimens. FNEs and NEOs were more frequently seen in the antero-superior and postero-superior zones. Four specimens had abundant vascularity and disorganised architecture of FNEs in the deeper zones of the antero-superior quadrant suggestive of a healed tear. Myofibroblasts were present in abundance in all the labral specimens and were distributed uniformly throughout all labral zones and depth.

**Conclusions:**

The current study shows that the human acetabular labrum has abundant FNEs and NEOs. These are more abundant in the antero-superior and postero-superior zones. The labrum, by virtue of its neural innervation, can potentially mediate pain as well as proprioception of the hip joint, and be involved in neurosecretion that can influence connective tissue repair.

## Background

The acetabular labrum is a complex fibro-cartilaginous structure that is attached to the acetabular rim [1]. It has long been believed to be a site of pain generation in various pathologies of the hip joint [2,3]. In young patients with femoro-acetabular impingement (FAI), the acetabular labrum has been shown to be affected by the impingement process [4]. However, most of the patients remain asymptomatic despite structural abnormalities in their hips, preventing early diagnosis and intervention [5]. Also, in patients with hip osteoarthritis, there is a lack of strict correlation between the degree of arthritic changes on hip radiographs and patient symptoms.

Over the last decade, hip arthroscopy has emerged as an important procedure for addressing labral pathology [6,7]. Despite advancements in techniques of labrum repair, it is still unclear whether labral repair is a better long-term treatment than partial labrectomy for a labral tear [8]. For example, a recent review on the management of labral tears did not support routine labral repair over labral debridement [9]. There is a paucity of well-performed clinical studies, as well as few studies that clearly describe the neurohistology of the labrum. Hence, the source and mechanism(s) of hip pain remain unknown, which contributes considerable uncertainty for physicians and surgeons who must manage labral tears.

A better understanding of the innervation of the human labrum may help address many questions related to symptomology and management of various hip pathologies. Very few studies in the English literature have investigated the sensory innervation of the human labrum [10-13]. Although these studies have identified free nerve endings (FNE) and sensory nerve end organs (NEO) in the human labrum, there is limited information in the literature regarding the distribution of these neurosensory structures in the specific zones of the labrum.

The aim of the current study was to evaluate the innervation of the acetabular labrum in the various zones and to understand its potential role in nociception and proprioception in hips with labral pathology.

## Methods

Following institutional review board approval from the University of Calgary office of medical bioethics, an informed consent was obtained from all the patients undergoing total hip arthroplasty (THA) or hip resurfacing. Twenty labrums were excised intraoperatively from the acetabular rim with the aid of a scalpel. Care was taken to resect a single large segment of labrum. Any specimen not resected in toto because of significant deficiency of the labrum in any part was discarded. Labrums having calcific deposits were also discarded.

**Figure 1:**
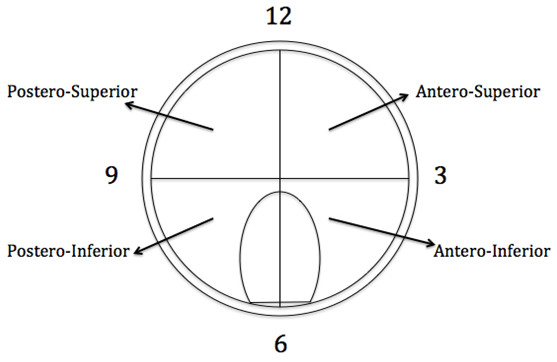
**Diagrammatic representation of histological zones of labral specimens (Right hip here).** Adapted from Gerhardt et al. [12].

After the harvest, all the specimens were tagged by a suture placed at the edge of the antero-inferior segment of the labrum (Figure 1). The specimens were then immediately immersed in normal saline and transported to the lab. These were cut into 5-7 mm blocks from different quadrants of labrum and embedded in Optimum Cutting Temperature (OCT) medium and snap frozen. The specimens were then stored at minus 80°C for at least 24 hours. Cryosections were cut at a thickness of 10 μm on a cryostat (Microm HM500 OM), mounted on glass slides, fixed in cold paraformaldehyde, and stored at minus 20°C. Sections were stained using Hematoxylin and Eosin (H and E). Serial sections were stained by indirect immunoperoxidase using a rabbit polyclonal antibody directed against S-100 protein (Dako Canada Inc.), which is widely expressed by cells of the nervous system. The detection system included biotin-conjugated anti-rabbit IgG, streptavidin-conjugated horseradish peroxidase, and diaminobenzidine as a chromogen. Absence of primary antibody served as a negative control. Slides were examined under the light microscope (Zeiss Axioskop2, 63X, 1.4NA objective) using differential-interference contrast illumination.

An analysis of the distribution of neurosensory structures in the labrum involved dividing the labrum into four quadrants (zones) using a clock face pattern: Antero-superior (AS), Postero-Superior (PS), Antero-Inferior (AI) and Postero-Inferior (PI) as shown in Figure 1.

## Results

A total of 20 specimens were harvested for evaluation. The mean age of these patients was 60.5 (range 42–78) and the primary diagnosis in all was symptomatic end stage osteoarthritis of hip.

**Figure 2:**
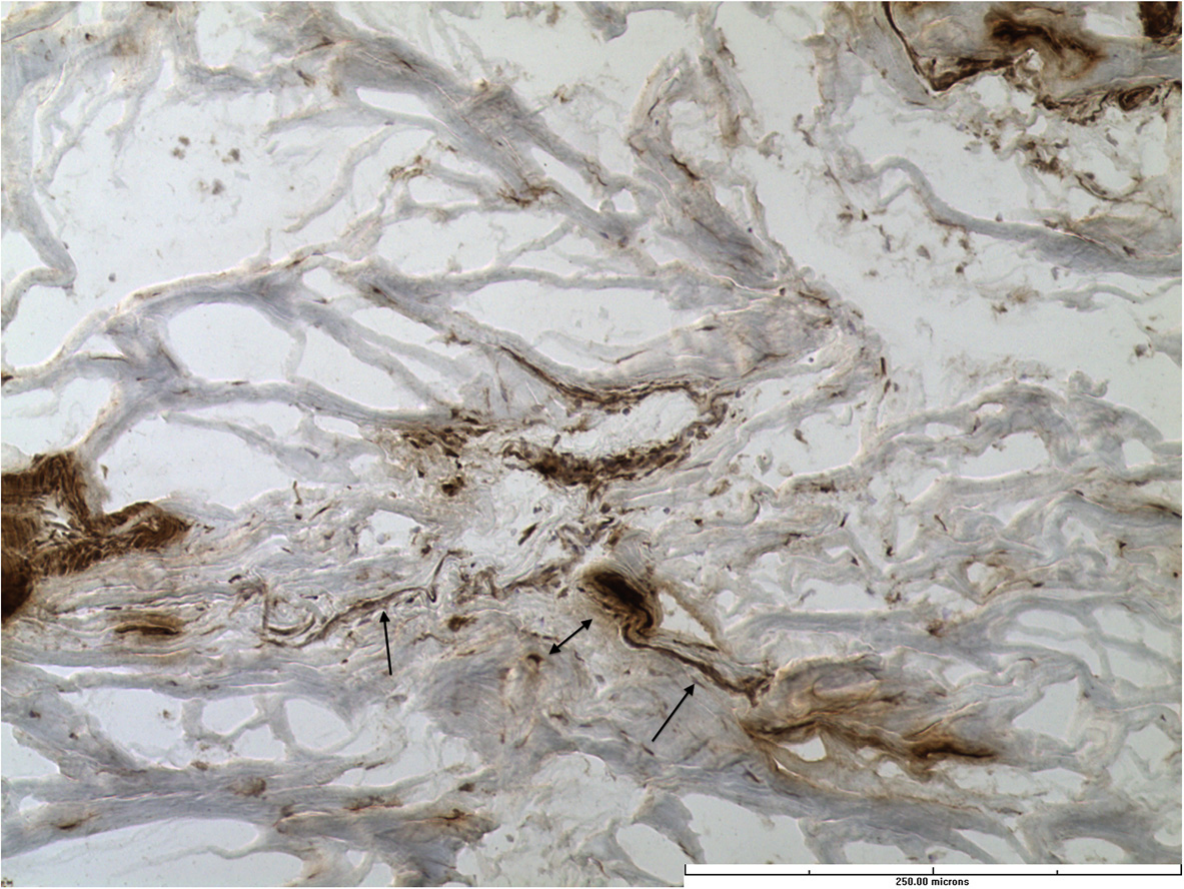
**Free nerve ending (single head arrow) and Ruffini corpuscle (double headed arrow).** (Immunohistochemistry stain for S-100 protein, x20).

**Figure 3:**
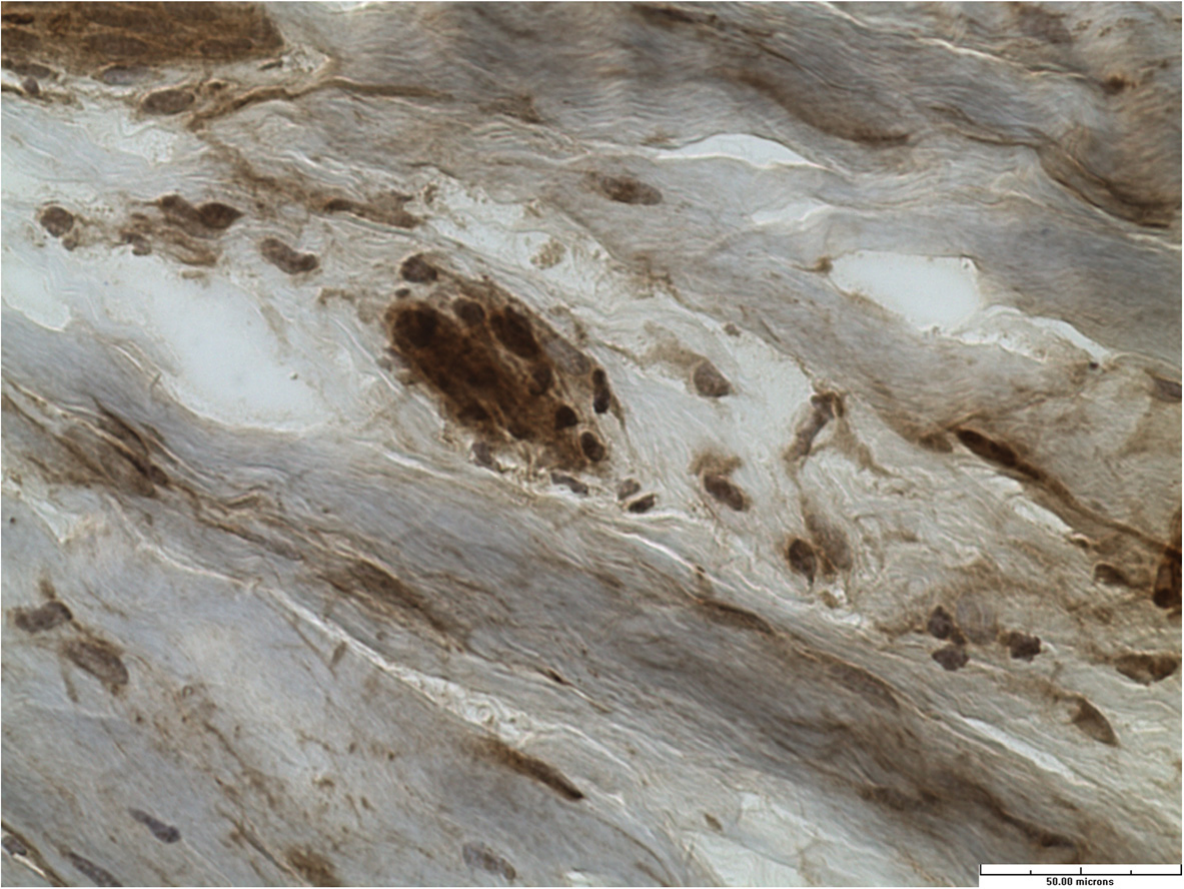
Golgi-Mazzoni corpuscle (immunohistochemistry stain for S-100 protein, x40).

**Figure 4:**
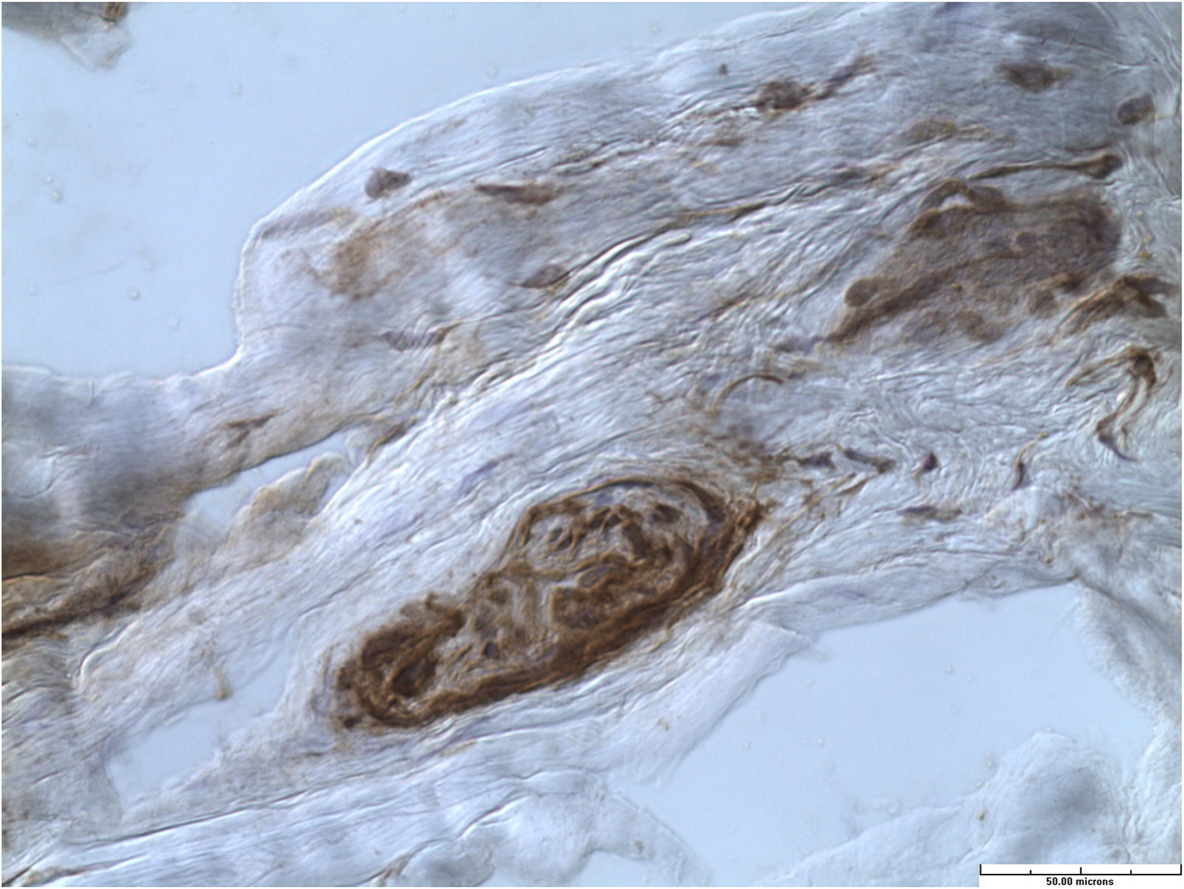
**Vater-Pacini corpuscle.** (Immunohistochemistry stain for S-100 protein, x40).

All specimens had abundant free nerve endings (FNE). These were seen predominantly superficially and on the chondral side of the labrum. In addition, predominantly three different types of nerve end organs (NEO) were identified in all twenty specimens. These included Vater-Pacini, Golgi-Mazonian and Ruffini corpuscles. The articular corpuscles (Krause Corpuscles) were not consistently seen and were identified in only 2 specimens. Representative histological sections with specific NEOs are shown in Figures 2, 3 and 4.

Although difficult to quantify precisely, FNEs were clearly more abundant in the AS and PS zones of the labrum as compared to the AI and PI zones. NEOs were also more frequently seen in the AS and PS zones. All the specimens had one or more NEOs in AS and PS zones. However, NEOs were identified in only 20-40% of specimens in the AI (8 specimens) or SI (4 specimens) zones. These findings have been summarized in Table 1.

**Table 1:** Distribution of FNEs and NEOs in different quadrants of labrum

**Zones**	**AS**	**PS**	**AI**	**PI**
Superficial	20	20	8	4
Deep	4 (FNEs only)	0	0	0

**Figure 5:**
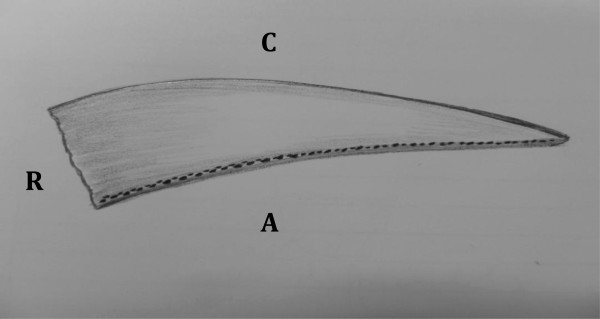
**Schematic representation of FNE and NEO within the substance of the labrum (Dots) seen mainly at the articular side of the labrum.** R: Acetabular rim, A: articular (chondral) side of the labrum, C: Capsular side of the labrum.

There were no appreciable differences in the innervation based on patient age. FNEs and NEOs were predominantly distributed in the superficial layer and on the articular (chondral) side of the labrum. These extended from the junction of the capsular and articular zone of the labrum towards the chondro-labral junction (in Figure 5).

**Figure 6:**
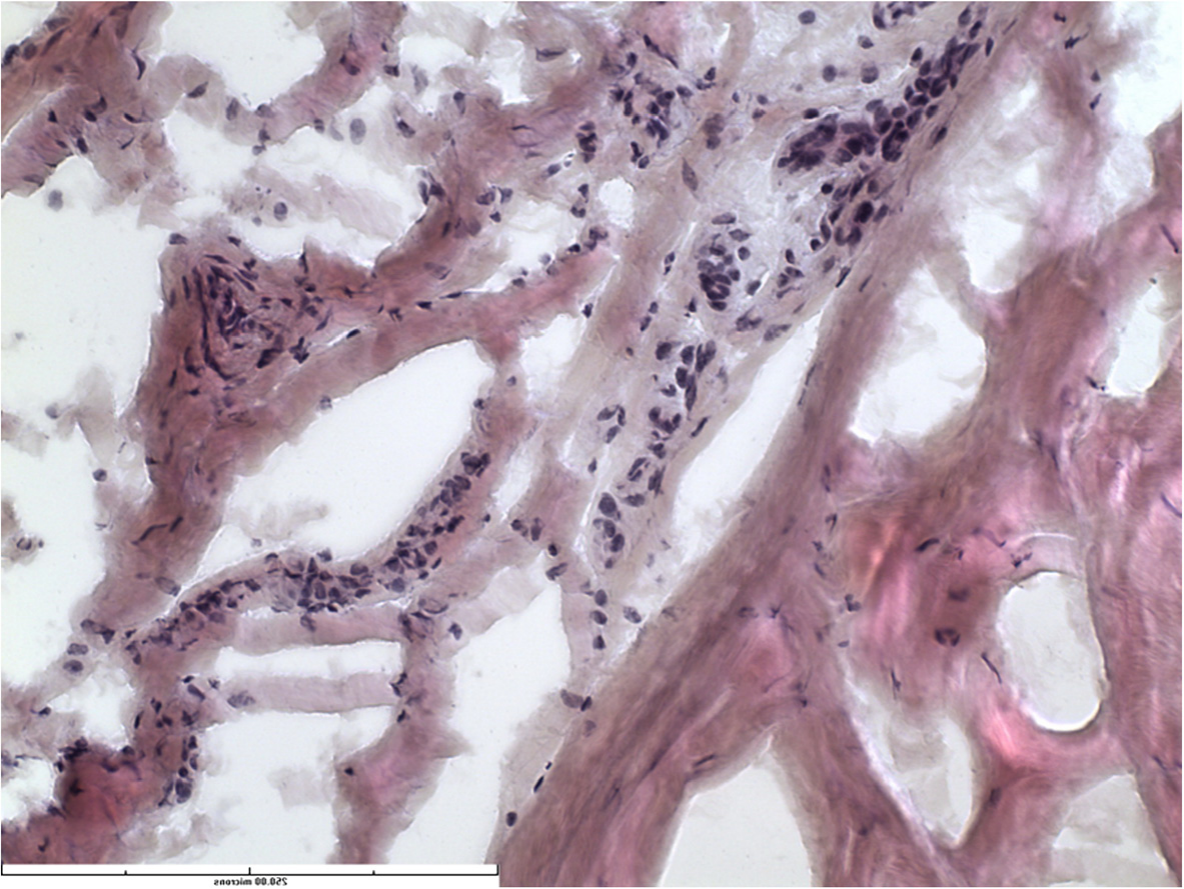
Section from midsubstance of the labrum showing increased vascularity (HE stain x40).

**Figure 7:**
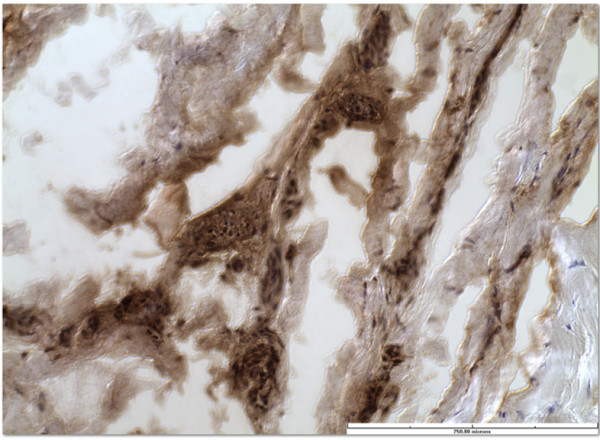
Free nerve endings after immunohistochemistry stain for S-100 protein, x40.

While the FNEs and NEOs were identified in the superficial region of all the labral specimens, these were seen in the deeper layers in only 4 specimens of labrum and only in the antero-superior zones of these specimens. In all these 4 specimens, the FNEs were seen running across the substance of the labrum. Further, these FNEs were less well organized, and had a more radial and oblique arrangement. Interestingly, an abundant vascularity (not visible in any other layer or any other specimen) accompanied the FNEs in these 4 specimens (Figures 6 and 7).

**Figure 8:**
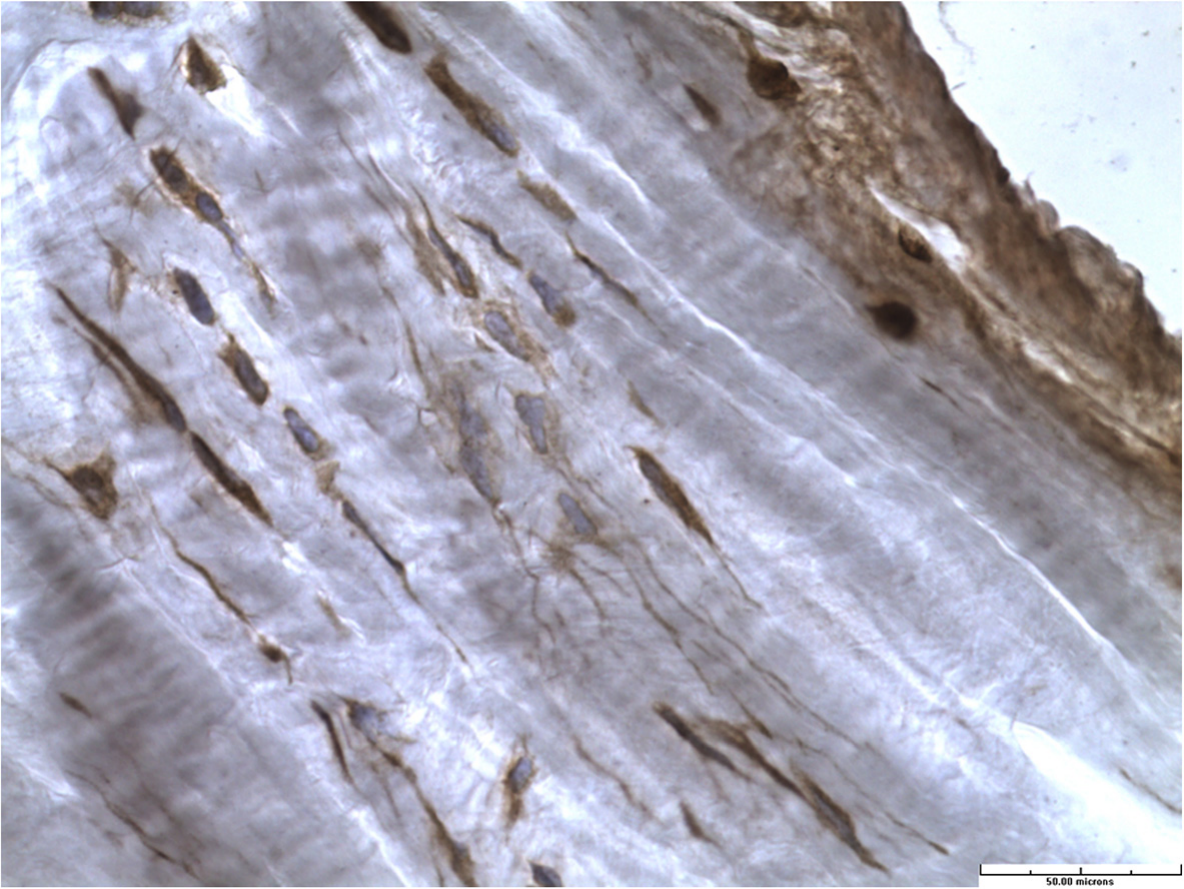
Fibroblast with elongated cell processes (immunohistochemistry stain for S-100 protein, x40).

Fibroblasts were present in abundance in all the labral specimens and were distributed uniformly throughout all labral zones and depth. These fibroblasts had elongated cell processes that extended into the extracelluar matrix (Figure 8).

## Discussion

The most important finding of this study was a consistent demonstration of FNEs and sensory NEOs in all the labral specimens with a higher prevalence in the antero-superior and postero-superior zones of the labrum. The presence of both FNEs and NEOs in the human labrum as demonstrated by the study clearly shows that the human labrum plays a role not just in transmitting nociception (through FNEs), but also providing proprioception to the hip joint (through NEOs).

To the best of our knowledge, this is the second study in the literature after the study by Gerhardt et al. [12] describing a detailed zone wise distribution of the innervation of the human acetabular labrum. Only 4 studies in the English literature have studied the innervation of the human labrum. These are summarized and compared with the current study in Table 2.

**Table 2:** Comparison with previous studies dealing with innervation of human acetabular labrum

**Authors**	**Year**	**No. of specimens**	**Mean age (yrs)**	**Technique**	**Results**
Kim and Azuma [10]	1995	23 cadaveric specimens	64.8	Suzuki’s Silver stain and Immunohistology for S-100	FNEs and NEOs in all specimens. More numerous in antero-superior zone in a single specimen that was used to evaluate zone-wise distribution on electron microscope
Shirai et al. [11]	2009	10 specimens harvested during THA.	-	Immunohistology for Protein Gene Product (PGP) 9.5 and TumorNecrosis Factor (TNF) alpha	Positive immunoreactivity in the weight bearing regions of OA patients. Negative in nonweightbearing region of OA patients. Negative in all the specimens from ONFH or NOF fracture group
		3 groups: Osteoarthritis (OA), Osteonecrosis Femoral Head (ONFH) and fracture neck of femur (NOF)			
Gerhardt et al. [12]	2012	8 hips of cadavers.	76.5	Modified Gold Chloride Staining	Highest concentration in anterior zones of labrum and closer to chondro-labral junction
		10 sites of specimens from each hip.			
		Evaluated hip capsule, transverse acetabular ligament and ligamentum teres apart from labrum			
Hawersath et al. [13]	2013	44 labral specimens from 57 patients. Evaluated antero-superior labrum only, along with ligamentum teres and capsule	55.6	H and E, Immunohistology	Evaluation of antero-superior labrum only. Pain-associated FNEs predominantly at the base of labrum (acetabular attachment), decreasing in the periphery. Highest concentration in the middle third (1 o’clock to 2 o’clock).
Current study	2013	20 specimens harvested during THA or hip resurfacing	60.5	H and E, and Immunohistology for S-100	FNEs and NEOs in all the specimens. Higher concentration in antero-superior and postero-superior zones as compared to antero-inferior and postero-inferior zones. Higher concentration superiorly and on the articular (chondral) side than the capsular side.

Labral innervation primarily comes from a branch of the nerve to the quadratus femoris as well as the obturator nerve [14]. The ramified nerve endings, which primarily play a role in nociception, have been identified within the substance of the labrum [10,15]. The various sensory NEOs identified are primarily receptors for pressure, deep sensation and temperature; and play a role in the proprioceptive feedback [8,10,12].

In FAI, the labrum gets crushed between the acetabular rim and femoral neck with different patterns of involvement in cam and pincer lesions [4]. On examination, the impingement test (flexion, internal rotation and adduction) primarily stresses the antero-superior aspect of the labrum, and pain with this maneuver may well be originating from abundant nerve endings in this zone. Labral tears are also more common in the antero-superior zones of the labrum in FAI [16]. The presence of abundant free nerve endings in these zones transmitting nociception clearly explains the symptoms of acute pain with labral tears.

Wyss et al. [17] reported that patients with FAI presenting with hip pain had actually noticed diminished mobility in the affected hips long before the pain appeared. This clinical result is difficult to reconcile based on our anatomical findings regarding innervation of the hip labrum. Considering the fact that the antero-superior zone of the labrum is the one that is most frequently involved in FAI and this is the zone in our study that is found to have the most abdundant FNEs carrying nociception, there must be some other factors (not explained by our study) that regulate pain sensation in a hip joint with FAI.

The neural and vascular anatomy of the hip labrum differs from that of knee menisci. The anterior and the posterior horns of menisci in the knee have a rich vascularity associated with numerous nerve endings [18]. However, this is not the case with the human labrum, as seen in the current study. The nerve endings were abundant in the superficial zones of the acetabular labrum, which were largely avascular. Kelly et al. [19] in a cadaveric study using the Spalteholz technique found the vascular supply to the acetabular labrum relatively poor especially on its articular side where we found the maximum concentration of FNEs and NEOs. Seldes et al. [20] also noted a relative lack of vascularity in the acetabular labrum in their histologic study. These authors found only a group of small vessels travelling circumferentially around the labrum at its attachment site on the outer surface of the bony acetabular extension (labrocapsular junction).

An interesting finding in the current study was the presence of loosely organised FNEs in the deeper layers of the labrum in the antero-superior zones of 4 of the specimens. These nerve fibers had more oblique and radial orientation, and were associated with abdundant vascularity. These findings have not been described by any of the previously published studies [10-13]. We believe that these represent areas of healed tears in the antero-superior zones and the nerve fibers may have a vasomotor function in these cases.

Another important finding in the current study was the presence of microfilaments rich fibroblasts in the labrum giving them the appearance of myofibroblasts. These were found to be dispersed uniformly through the acetabular labrum. These myofibroblasts are specialized contractile fibroblasts, which contribute to the reconstruction of injured tissue by secreting extracellular matrix and exerting high contractile forces that help in healing of many tissues including open wounds and avascular tissues like the cornea [21-23]. Theoretically, these myofibroblasts could play a role in healing of labral tears in the setting of poor vascularily in the acetabular labrum. Seldes et al. [20] in their histological study showed that neovascularization occurred within the substance of the labrum following a labral tear. Neovascularisation was also seen within the area of the tear. They suggested synovial or the subchondral bleeding responsible for this phenomenon. Whether the native myofibroblasts of the labrum play any role in this process remains unanswered.

The strength of the current study included larger numbers than the previously published study. Kim and Azuma [10] evaluated the zonal distribution in only one specimen. Also, such a detailed assessment of the zones of the labrum, including findings suggestive healed tears and presence of myofibroblasts has not been described previously by any of the authors. Although Hawesath et al. [13] harvested 44 labrum specimens; they predominantly evaluated the nociception in the antero-superior labrum only.

One clear limitation of the study was evaluation of the labrum harvested from hips with end stage symptomatic osteoarthritis. Although all the specimens had consistent variation in density of FNEs and NEOs, this may not be truly representative of a labrum from a non-pathological hip or a labrum from a young adult with FAI with or without a labral tear.

Nevertheless, the study provides useful new information regarding the innervation of the hip labrum. Our study shows that human acetabular labrum has abundant FNEs and NEOs. These are more abundant in the antero-superior and postero-superior zones. The labrum, by virtue of its neural innervation, can potentially mediate pain arising in the hip joint, mediate proprioception of the hip joint, and be involved in neursecretion that can influence connective tissue repair [24]. Healing of labral tears can occur spontaneously, and the abundant myofibroblasts in the labrum may play a role in the process.

## Conclusion

In conclusion, from a clinical point of view, arthroscopic labral debridement (partial labrectomy) may lead to more predictable pain relief than labral repair because of excision of pain-mediating FNEs in the torn labrum. However, labral repair might be a better option in the long run as it preserves the proprioceptive function of the hip joint and this might in turn increase the longevity of a pathological hip.

## Competing interests

The authors on this study wish to declare that they have no competing interests.

## Authors’ contributions

Conception, design of study, preparation and submission of application for ethics: JP, AA, PR, RG. Acquisition of data: JP, AA, PR. Preparation of specimens, analysis and interpretation of data: AA, JM, DP, KB. Manuscript preparation and revisions: JP, KB, AA, PR, RG, JM. Final Manuscript edit and approval: JP, AA, PR, RG, KB, DP, JM.

## Pre-publication history

The pre-publication history for this paper can be accessed here:

http://www.biomedcentral.com/1471-2474/15/41/prepub
